# Academie De Medecine

**Published:** 1889-05

**Authors:** 


### ACADEMIE DE MEDECINE.

### Antipyrin in Glycosuria.

At the last meeting of the Académie de Médecine a discussion on the action of antipyrin in glycosuria occurred. M. Panas related two cases which he considered important. The first was that of a man of 38, who was affected with bilateral cataract for two years previously, excreted in the twenty-four hours over an ounce and a half of sugar. Finding that little or no relief was given him at his own home, he entered the hospital, and placed himself under the care of M. Panas. A rigorous dietetic treatment was immediately adopted, with the effect that the quantity of sugar diminished to half an ounce, but that amount persisted, in spite of all treatment. The patient consented to the removal of one of his cataracts; M. Panas decided to operate. The result was a perfect success, in spite of the persistent glycosuria. He then left the hospital for some time, and returned at the beginning of the present year. His urine then contained nearly two ounces of sugar in the twenty-four hours. The usual *regime* for diabetes was ordered, and in a few days the quantity of sugar fell to one ounce. After trying iodide of sodium, bromide of potassium without effect, M. Panas ordered three grammes of antipyrin a day, and in a week no trace of sugar was found, although the patient had eaten ordinary bread. The second cataract was removed, and when the man left the hospital one drachm of sugar was found, but he had two days previously been left without the antipyrin. The second case was that of a woman, aged 73, diabetic for six years. She had also double cataract, and the quantity of sugar was but little inferior to that of the first case. The administration of three grammes of antipyrin daily reduced it to sixty grains, and finally to twelve grains. In conclusion he said that he considered antipyrin to have a very prompt antiglycogenic action, that it succeeds where all other treatment failed, and that the dose should be twenty grains three times a day.

M. G. See said that he treated eighteen patients by antipyrin, and his experience was that where the sugar did not exceed in quantity three ounces in the twenty-four hours that drug effected frequently a complete cure. Not only does the thirst, polyuria, glycosuria, etc., disappear, but also the different nervous and cutaneous troubles.

M. Dujardin said that fully a year ago he had the good effects of antipyrin.

M. Robin said that for eighteen months he treated diabetes by antipyrin, and he found that it acted with energy on glycosuria, but he did not meet with one case of complete cure. As to the method of administering it, he thought it would be well to associate with it a little bicarbonate of soda, and not to continue it longer than ten or twelve days, as its constant use would likely produce albuminuria.

